# The Baltimore College of Dental Surgery

**Published:** 1882-06

**Authors:** 


The Baltimore College of Dental Surgery.—In consequence of the resignation of Professors Gorgas and Harris from the faculty of this institution, certain changes have been made in its management, which tend to combine it with the College of Physicians and Surgeons of Baltimore. Professors Latimer, Bevan, Coskery, and Gundry, of the latter College, have been elected to the chairs of Physiology, Anatomy, Oral Surgery, and Materia Medica and Therapeutics respectively in the dental school. Many of the lectures will be the same to the students in both Colleges, and the students of the dental school will have the privilege of attending the lectures in the medical school. Graduates of the Baltimore College of Dental Surgery will also be permitted to come forward for the degree of Doctor of Medicine in the College of Physicians and Surgeons, upon attending one course of lectures after receiving the degree of D.D.S. Students who intend to avail themselves of this advantage, however, are required to notify the dean of the medical school when beginning their second course in the dental school.

